# Macrophyte Potential to Treat Leachate Contaminated with Wood Preservatives: Plant Tolerance and Bioaccumulation Capacity

**DOI:** 10.3390/plants9121774

**Published:** 2020-12-14

**Authors:** Emmanuelle Demers, Margit Kõiv-Vainik, Sara Yavari, Michel Mench, Lilian Marchand, Julie Vincent, Chloé Frédette, Yves Comeau, Jacques Brisson

**Affiliations:** 1 Département de Sciences Biologiques, Institut de Recherche en Biologie Végétale, Université de Montréal, 4101 Sherbrooke East, Montreal, QC H1X 2B2, Canada; emmanuelle.demers@gmail.com (E.D.); sarayavary2020@gmail.com (S.Y.); jooolievincent@gmail.com (J.V.); chloe.fredette@quebecvert.com (C.F.); jacques.brisson@umontreal.ca (J.B.); 2 Department of Geography, Institute of Ecology and Earth Sciences, University of Tartu, 46 Vanemuise St., 51003 Tartu, Estonia; 3 University of Bordeaux, INRAE, BIOGECO UMR, 1202, Bat B2, Allée G. St-Hilaire, 33615 Pessac, France; michel.mench@inrae.fr; 4 SUEZ-Le LyRE, Research and Innovation Center, 15 Avenue Léonard de Vinci, 33600 Pessac, France; lilian.marchand@suez.com; 5 Department of Civil, Geological and Mining Engineering, Polytechnique Montréal, 2500 Polytechnique Road, Montreal, QC H3T 1J4, Canada; yves.comeau@polymtl.ca

**Keywords:** chromated copper arsenate, chlorinated phenols, phytoremediation, constructed treatment wetland, *Typha angustifolia*, *Phalaris arundinacea*, *Phragmites australis*

## Abstract

Pentachlorophenol and chromated copper arsenate (CCA) have been used worldwide as wood preservatives, but these compounds can toxify ecosystems when they leach into the soil and water. This study aimed to evaluate the capacity of four treatment wetland macrophytes, *Phalaris arundinacea*, *Typha angustifolia*, and two subspecies of *Phragmites australis*, to tolerate and treat leachates containing wood preservatives. The experiment was conducted using 96 plant pots in 12 tanks filled with three leachate concentrations compared to uncontaminated water. Biomass production and bioaccumulation were measured after 35 and 70 days of exposure. There were no significant effects of leachate contamination concentration on plant biomass for any species. No contaminants were detected in aboveground parts of the macrophytes, precluding their use for phytoextraction within the tested contamination levels. However, all species accumulated As and chlorinated phenols in belowground parts, and this accumulation was more prevalent under a more concentrated leachate. Up to 0.5 mg pentachlorophenol/kg (from 81 µg/L in the leachate) and 50 mg As/kg (from 330 µg/L in the leachate) were accumulated in the belowground biomass. Given their high productivity and tolerance to the contaminants, the tested macrophytes showed phytostabilization potential and could enhance the degradation of phenols from leachates contaminated with wood preservatives in treatment wetlands.

## 1. Introduction

Pentachlorophenol (PCP) and chromated copper arsenate (CCA) are the wood preservatives most commonly used in North America to preserve wooden utility poles [1]. PCP is an organic preservative containing chlorinated phenols and hydrocarbon impurities such as dibenzo-dioxins/furans (PCDD/Fs). CCA is an inorganic compound composed of arsenic (As), chromium (Cr VI), and copper (Cu). PCP and CCA are potentially toxic to human health and ecosystems when released into the environment [2]. In utility pole storage sites where the treated poles are exposed to atmospheric precipitation, PCP and CCA can leach into the soil and flow freely to the surface and groundwater.

PCP and PCDD/F are relatively persistent in soil and undergo slow natural attenuation [3]. Their partial microbial biodegradation and photodegradation increase the presence of various chlorinated phenols in the leachate that can generate high toxicity and mutagenic effects in living organisms [4]. There is some evidence that aquatic plants play an important role in the fate of PCP through uptake and elimination kinetics [5], but the toxic effect and fate of different forms of chlorinated phenols in plant tissues has not been thoroughly studied [6].

The As, Cr, and Cu present in the leachate of CCA-treated utility poles can take different forms: arsenate (As V) and arsenite (As III); trivalent Cr and hexavalent Cr; and inorganic Cu (Cu^2+^) and Cu bound to dissolved organic matter, respectively [7,8,9]. The toxicity of As arises from the chemical similarity between arsenate (AsO_4_^3−^) and phosphate (PO_4_^3−^), which can interfere with phosphate metabolism in living organisms [10]. The toxic effect of Cr (VI) is related to its strong oxidative potential and the production of free radicals in cells [11]. Although Cu is an essential element for living organisms, it causes toxicity at high concentrations because it generates oxidative stress in cells [12].

The potential phytotoxicity of As (III and V), Cr (VI), and Cu due to excessive root exposure has been described in many publications [13,14,15]. The toxic influence of individual PCP and CCA in soil and water on plant growth has been studied most often [8,16]. However, co-existence of PCP and CCA in soil and water is of great concern due to their interactions, which complexify their bioavailability and toxicity in the environment [17]. In excess, metal(loid)s can inhibit microbial biodegradation of organic pollutants in soil. Organic pollutants may in turn combine with metals and form less bioavailable compounds [18]. According to Shanker et al. [19], Cr (VI) phytotoxicity includes changes in the germination process and biomass growth (especially inhibition of root growth [20]), and can have deleterious effects on photosynthesis, water relations, and mineral nutrition. For example, Cr (VI) resulted in phytotoxicity for all plant species investigated in Ranieri and Gikas [21]. Cu toxicity can lead to failure in photosynthesis, thereby affecting plant growth and survival [22]. Considering the toxicity and complexity of wood preservative leachates, applying an effective cleanup strategy prior to their release into the environment is imperative.

Phytoremediation technologies use living plants to clean up air, soil, and water polluted with harmful contaminants. A variety of processes facilitated by plants are used for treating environmental problems [23]. Among the different phytoremediation technologies used to treat leachate, treatment wetlands (TWs), which rely on the natural properties of plants and their associated microorganisms, can provide a range of benefits in contaminant treatment [24]. They are known to be cost-effective and easily managed treatment systems in terms of operation, maintenance, and services provided [24,25]. Macrophytes are the principal component of TWs and have direct and indirect roles in contaminant treatment. Macrophytes have been suggested for pollution control through phytoremediation because of their ability to accumulate metal(loid)s and nutrients as well as enhance the degradation of organic pollutants [22,26,27,28,29,30,31,32]. Contaminants can be directly absorbed by macrophytes or adsorbed to their roots. Inside the plants, contaminants can be accumulated in plant tissues (bioaccumulation), degraded by the enzymes’ activities and endophytes (phytodegradation), or released as volatile forms from the aerial parts (phytovolatilization) [26]. On the root surface, the contaminants can be deactivated by the root exudates or the cell walls’ pectin compounds (phytostabilization) [33]. In addition to immobilizing contaminants in their tissue, plants may also contribute to phytostabilization processes such as stabilization of the substrate, reduction of current velocity, provision of surfaces for periphyton attachment, and release of oxygen [34]. Indirectly, macrophytes stimulate the presence of degradative microorganisms by providing root exudates (rhizoremediation) and/or assist in the co-precipitation of contaminants with oxide compounds by diffusing oxygen around their rhizosphere [35].

Although macrophytes have been used for PCP and CCA treatment in TWs, the removal efficiency of various plant species requires further study. Šíma et al. [36] reported high As, Cr, and Cu removal efficiencies (236%, 64%, and 70%, respectively) in a TW planted with *Phragmites australis*. Kadlec and Zmarthie [37] observed a lower removal efficiency of As (29%) in a leachate treatment TW system with cattails (*Typha latifolia*). Zhao et al. [38] demonstrated a high PCP removal from contaminated sediments planted with *Phragmites communis* Trin (90%), *Theileria orientalis* (99%), and *Scirpus validus* (99%). Results of a study by Lévesque et al. [39] showed that a TW planted with *P. australis*, followed by a wetland planted with willows (*Salix miyabeana* SX67), were efficient in removing PCP (98%), arsenic (99%), chromium (99%), and copper (>99%). Furthermore, Frédette et al. [40] demonstrated that *Salix miyabeana* SX67 showed a capacity to bioaccumulate all analyzed contaminants (PCPs and CCA) even at low concentrations and showed potential to translocate PCDD/Fs (polychlorinated dibenzo-dioxin/furan congeners) and Cu to aerial plant parts.

Additional information is needed to ensure optimal plant selection when TW systems are used to address specific mixtures of toxic pollutants. It is important to identify wetland plant species that are tolerant to wood preservatives and have beneficial characteristics that would contribute to the reduction of PCP and CCA from polluted water (e.g., through plant uptake and immobilization by enhancing microbial processes in TW substrate and water).

Furthermore, most previous phytoremediation studies have primarily concentrated on determining the effect and/or removal of particular organic or inorganic compounds (e.g., [5,14,15,19,21,22,30,33,38,41]), while others have examined similar issues with mixed contaminants (e.g., [42,43,44]). Overall, less attention has been devoted to the synergic effect of contaminants on plant physiology (e.g., [40,45,46]). When considering real life field conditions, plants are in fact generally exposed to a mixture of different organic and inorganic contaminants (e.g., [39,47]). Further study of the effect of these complex contaminated waters on TW plant species and the removal potential of these species is needed.

This study aimed to evaluate the tolerance and bioaccumulation potential of four macrophyte species, *Phalaris arundinacea*, *Phragmites australis* subsp. *australis*, *Phragmites australis* subsp. *americanus*, and *Typha angustifolia*, when exposed to leachate contaminated with wood preservatives. To achieve this objective, a pot experiment was carried out to compare the performance of selected macrophytes in the presence of four concentrations of the studied contaminants in the leachate. The macrophytes tested in this experiment are frequently used in TWs for their high productivity and tolerance to highly polluted conditions [48,49]. Native to Eurasia, *P. australis* subsp. *australis* is the most widely used taxon in TWs worldwide, but is also considered highly invasive in North America and in other parts of the world [50,51]. Hence, for comparison purposes, we also tested its North American native counterpart, *P. australis* subsp. *americanus*, which has been shown to be equally promising for TWs [52] and two other common TW species.

## 2. Results

**Figure 1:**
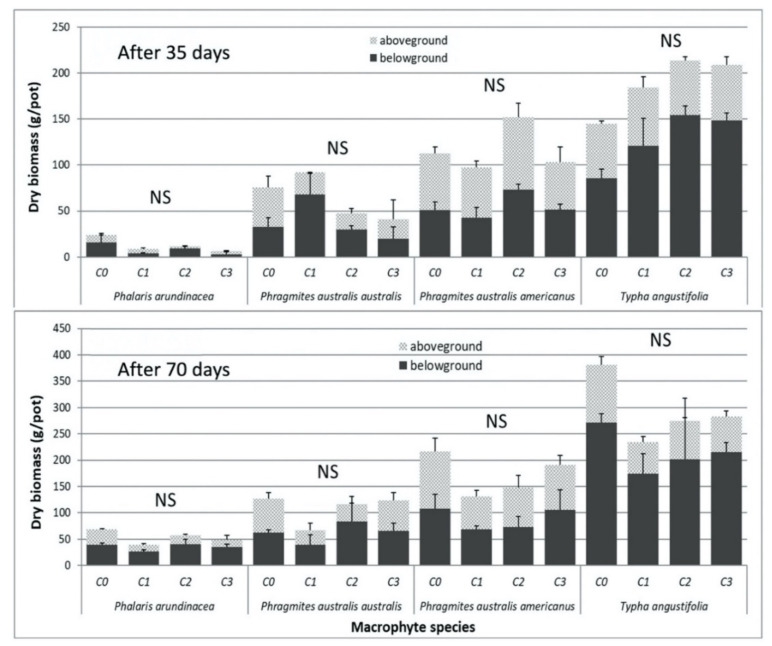
Mean (with standard error) above- and belowground dry biomass (g/pot) of the four macrophyte species exposed to four contaminant concentrations (**C0–C3**) in the leachates after 35 and 70 days. (**NS**) No significant difference between mean values across the treatments for a given sampling date of a plant species, for either total biomass or aboveground and belowground biomass considered alone, according to Tukey’s test, α < 0.05.

There were no visual signs of phytotoxicity on the macrophytes tested and no statistical differences in the stomatal conductance and leaf chlorophyll content (measures determining health of the plants) between different leachate concentrations (Table S1). No significant differences were detected in above- or belowground biomass production or in the growth of aerial parts (i.e., height and number of stems) across the four macrophyte species in response to the contaminated leachates as compared to the control (C0) after 35 and 70 days of exposure (Figure 1, Table S1). However, at day 70, the average total biomass was slightly higher in the control treatments for these four macrophyte species (Figure 1).

**Figure 2:**
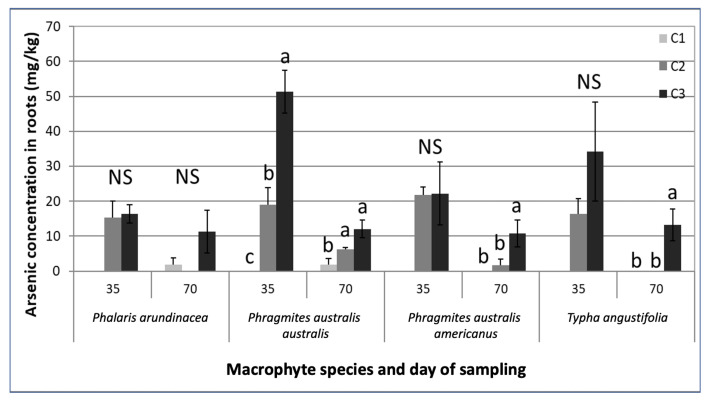
Mean (with standard error) arsenic concentration (mg/kg of DW) in the belowground parts of the four macrophyte species exposed to the contaminated leachates (concentrations **C1–C3**) after 35 and 70 days. For a given sampling date, mean values with identical letters were not significantly different according to the Tukey HSD test, *α* < 0.05. (**NS**) No significant difference between mean values across the three treatments for a given sampling date.

Chromium and Cu concentrations were below detection limits in above- and belowground plant parts for all species. We found no detectable As in the shoots, but As was present in roots and rhizomes of all species (Figure 2, Table S2a). The concentrations in the plant tissue were greater with increasing leachate concentrations for all species except *P. arundinacea* after 70 days (Figure 2, Table S2b). Only *P. australis australis* showed a significant difference in As in belowground plant parts in relation to contamination level at day 35.

**Figure 3:**
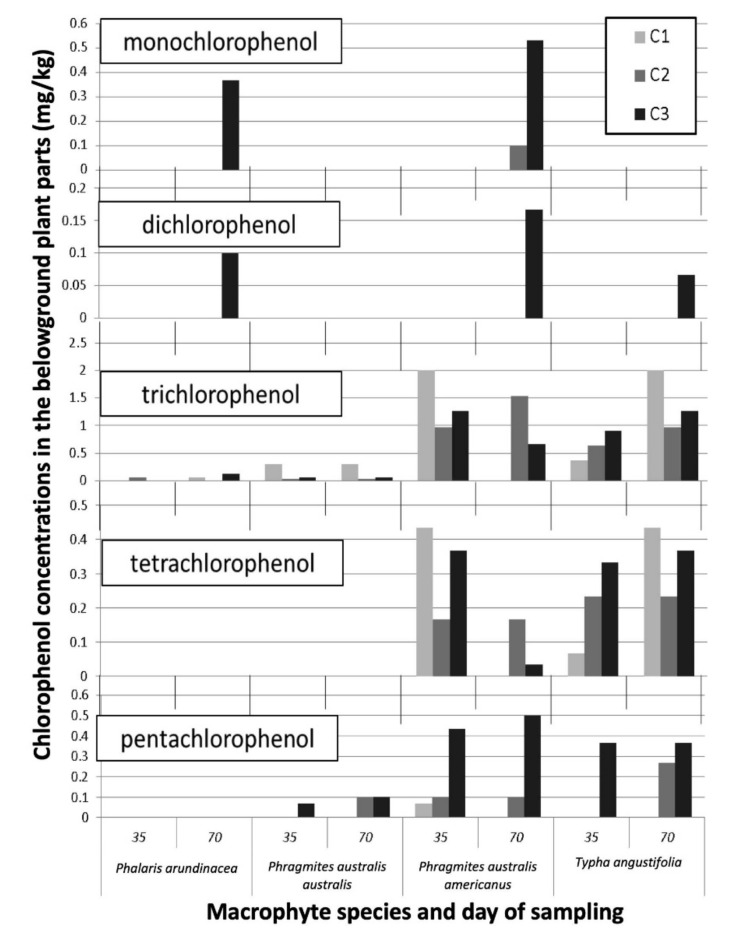
The chlorinated phenol concentrations (mg/kg DW) in the belowground dry biomass of the four macrophyte species exposed to the contaminated leachates (concentrations **C1–C3**) after 35 days and 70 days. No standard errors were calculated, or statistical inferences done due to the large number of samples with undetected levels of phenols.

We found chlorinated phenols in the belowground plant parts of all macrophyte species, but none were detected in the shoots (Figure 3). While the large number of samples with undetected phenols prevents us from making statistical inferences, a few strong patterns emerge from the qualitative examination of the results (Figure 3 and Table S3). First, tetra- and pentachlorophenols were detected in higher concentrations in the belowground plant parts as compared to the mono-, di-, and trichlorophenols. Second, there were apparent differences in accumulation between macrophyte species, with considerable concentrations of chlorinated phenols detected in the root tissues of *P. australis* subsp. *americanus* and *T. angustifolia*, but low levels of some phenols in *P. arundinacea*. Phenols were more often detected when the plants were exposed to higher contamination levels.

## 3. Discussion

### 3.1. Toxicity Effects on Plants

The fact that there were no signs of phytotoxicity and no significant differences in biomass production in relation to the contamination levels suggests that the selected macrophytes have the ability to resist the contamination levels of the experimental leachates within the range used in our study (20 to 4300 µg PCP/L; 83 to 890 µg As/L; 10 to 80 µg Cr/L; 25 to 400 µg Cu/L; Table 1). In findings consistent with this study, Dong et al. [53] reported that there was no difference in plant height and biomass of *T. angustifolia* when the plants were exposed to 100 µg Cr/L compared to the control (0 µg/L). Chandra and Yadav [54] observed no biomass reduction in *P. australis* and *T. angustifolia* grown in solution containing 2430 µg Cr/L and 48,000 µg Cu/L after 56 days of experiment. In contrast, *T. angustifolia* displayed stunted growth and necrosis symptoms at 400 µg As/L [55]. However, Bonanno et al. [56] showed that *T. angustifolia* tolerated sediment As concentrations up to 11,120 µg/kg. Here, the four macrophyte species survived well in the presence of up to 890 µg As/L. Plant phenological stage and vigor have been reported to influence the tolerance capacity of plants under high metal(loid) concentrations [57]. Although the experimental plants were exposed to a mixture of chlorinated phenols and metal(loid)s, no negative effects were observed on plant biomass. Similar results were obtained by Mills et al. [58], showing that *Salix* sp. “Tangoio” and *Populus* sp. “Kawa” tolerated soil contaminated with 250 mg PCP/kg, 3 mg As/kg, 18 mg Cr/kg, and 3.1 mg Cu/kg. Furthermore, in a mesocosm experiment, willow (*Salix miyabeana* SX67) proved to be tolerant to irrigation with raw leachate, with only leaf area decreasing with increasing leachate concentration, with maximum soil concentration of 0.3, 0.035, and 0.13 mg/kg of As, Cr, and Cu, respectively [40].

>Although not significant, the average total biomass was slightly higher in the control treatments for all four macrophyte species at day 70, which suggests that a longer exposure time might have shown a detrimental effect of the contamination developing over time. This pattern could also be the result of differences in contaminant concentrations in the leachates at both measurement times, especially higher arsenic concentration.

### 3.2. Bioaccumulation of Chromated Copper Arsenate (CCA)

We found no metal(loid)s in the aboveground portions of the plants, precluding the use of phytoextraction and plant harvesting as a remediation method [57,58]. The fact that no Cr and Cu was detected in above- or belowground plant parts was not surprising, considering their low concentration in the experimental leachates and the rather high detection limit we set for Cr and Cu in plant tissues (45 and 40 mg/kg DW, respectively). With a much higher Cr concentration in water, a significant accumulation of Cr was shown in other studies [20,21,59]. In Ranieri et al. [20], *P. australis* was able to accumulate 1910 mg Cr/kg DW into roots and 579 mg Cr/kg DW into stems. Furthermore, Rainier and Gikas [21] detected that exposure to Cr(VI) resulted in phytotoxicity for all tested plant species (e.g., *P. australis*, *Ailanthus altissima,* and *Salix viminalis*), expressed as a slowdown in root growth rate of up to 14% for *S. viminalis*, 24% for *P. australis*, and 57% for *A. altissima* compared to the control values. However, common reed and basket willow also showed total Cr removal of 56% and 70%, respectively.

Arsenic was detected in the belowground plant parts of all species, but there was no translocation to the aerial plant parts. A greater proportion of As in the root system of macrophytes has been reported in the literature [55,60,61]. *Phragmites australis* was shown to consistently accumulate more As in belowground parts than in the shoots (e.g., [60,62]. *Typha* spp. was reported to accumulate As primarily in roots and only minor amounts of As were translocated to shoots [63]. Higher levels of As in root tissue (232 mg/kg) of *Typha* sp. rather than shoots (17.2 mg/kg) were also detected by Dushenco et al. [55]. The greater presence of As in the root tissue can also be the result of adsorption of As on root surfaces. This process has been reported to be a critical step in phytoremediation of As by aquatic macrophytes. Macrophytes are able to form plaques of iron/manganese on their root surfaces that control the uptake and transfer of As in the plants by their affinity to As [64].

Our results regarding CCA removal by plants resemble those of other studies inasmuch as the highest amounts of metal(loid)s were accumulated or ad(b)sorbed by the belowground biomass of plants [21,27,29,56,59,60,65]. Ranieri et al. [20,21] demonstrated that *P. australis* and *S. viminalis* had highest Cr translocation affinity from the roots to stems and roots to leaves, respectively. Nonetheless, *P. australis* stored twice as much Cr into the belowground tissues than in aerial parts [20]. Bonanno [60] reported the same behavior and the general decreasing trend of metal(loid) content in *P. australis* was root > rhizome > leaf > stem.

As macrophytes primarily store metal(loid)s in belowground plant parts, they may contribute to immobilizing the toxin in a context of phytostabilization.

The concentrations in the plant tissue were greater with increasing leachate concentrations for all species except *P. arundinacea*. The belowground parts of the plants exposed to the C3 leachates had a higher As concentration than the plants exposed to C2 and C1 leachates. This tendency was significant for *P. australis* subsp. *australis* after 35 days and for *P. australis* subsp. *americanus* and *T. angustifolia* after 70 days of exposure. After 70 days, *P. australis* subsp. *australis* had significantly higher As concentrations in its belowground plant parts when exposed to the C3 and C2 leachates. A similar pattern occurred in *Typha* sp. roots in a study conducted by Dushenco et al. [55]. In their study, the highest As concentration was found in the roots of plants that were closest to the tailing discharge containing the highest As concentration.

### 3.3. Bioaccumulation of Chlorinated Phenols

The ability of macrophyte species to bioaccumulate chlorinated phenols in the roots has been documented by some authors [5,66]. In our study, chlorinated phenols were found in the belowground plant parts of all macrophyte species, but with large differences between species and types of phenols.

As the phenols are present in the soil water, sorption, binding, and absorption by root systems are the first possible processes of phenolic removal. Once absorbed, the phenols may be translocated by the transpiration stream to the aerial parts of plants [42] where they may accumulate, be metabolized, or volatilized [42]. Plant uptake and absorption is considered an important process in phenol removal by plants [6]. However, as chlorinated phenols are highly hydrophobic, diffusion to the sap and translocation to other plant parts remain generally low, and therefore they tend to remain largely in the roots, as our results suggest.

In our study, the most highly chlorinated phenols (tetra- and pentachlorophenol) were bioaccumulated in higher concentrations in the roots as compared to the low-chlorinated phenols. This greater bioaccumulation of the hydrophobic compounds can be mainly explained by their higher concentrations in the leachate (Table 1). Moreover, highly chlorinated phenols are more lipophilic. Thus, they have more affinity to root lipophilic molecules and might be sorbed more strongly by the root cells and diffuse more readily across cell membranes [67,68]. Much like CCA, phenols were more often detected in belowground plant parts when the plants were exposed to higher contamination levels. Similar results were observed with a woody plant, in which an increase in the exposure dose resulted in bioaccumulation of 2,4-dichlorophenols and 4-chlorophenol in *Salix viminalis* [69,70].

It has been repeatedly shown that uptake and translocation of organic pollutants may largely differ among plant species, so that conclusions regarding bioaccumulation by one cannot be applied to others [6]. In our study, we did find large differences between macrophyte species in phenol absorption, with higher concentrations of chlorinated phenols detected in the roots of *P. australis* subsp. *americanus* and *T. angustifolia*. *Phragmites* spp. can phytoextract xenobiotics to a significant degree and mainly accumulate these contaminants in its roots and rhizomes [71,72]. *Phragmites australis* is able to stimulate glutathione S-transferases and glucosyltransferases isoenzyme activity in the presence of xenobiotics that can assist in detoxifying these compounds prior to accumulation [73].

While plants may release degradative enzymes in the rhizosphere, the most important process of phenol removal remains microbial biodegradation. Plants may stimulate microbial activity by releasing oxygen or by exuding compounds that can serve as cometabolites to rhizospheric microorganisms [6]. Healthy plants with large and well-developed root systems may have a greater potential to contribute to this process, but more studies are needed to better understand the relation between plant functional traits and specific microbial strain activity [74].

## 4. Materials and Methods

### 4.1. Experimental Setup and Treatments

The pot experiment was conducted outside, at an industrial wooden utility pole storage site located in southern Quebec, Canada. This region has a humid continental climate with hot summers and cold winters. Average temperature in July and August 2013 at the closest weather station was 22 °C and 20 °C, respectively. Old utility poles are stored on shelf-like structures on top of an open leachate collection tank. Precipitation falling on the utility poles becomes contaminated with wood preservatives and the collected leachate is therefore contaminated with chlorophenols, arsenic, chromium (both Cr (III) and Cr (VI)), copper, and dioxins/furans. The volume of contaminated water and its contaminant concentrations largely vary depending on precipitation patterns, the number and state of the poles stored, how long they remain on sites, etc.

PCP levels in our first experimental batch (Table 1), even at the lowest C1 concentration, were higher overall than the measured values from leachates of our modeled industrial site in southern Quebec (i.e., 2.7 µg PCP/L [37]). However, metal(loid) concentrations for the C3 leachate were within the average values of the real leachate reported in the literature (e.g., 770 µg As/L, 130 µg Cr/L, 260 µg Cu/L [37]; and 260–530 µg As/L, 24–68 µg Cr/L, 160–180 µg Cu/L [43]), probably due to the sets of old wooden pole pieces we used in our maceration (Table 1). Still, the contamination levels in our experiment can be considered sufficiently high for evaluating plant resistance and performance.

To mimic rainwater contamination with common wood preservatives in this study, leachates with three concentrations (C1–C3) were prepared. For the C3 leachate, pieces of old wooden poles treated with PCPs or CCA were macerated in a 1 m^3^ tank filled with tap water for a one-month period. The C2 leachate was then obtained by diluting the C3 leachate with 50% tap water while the C1 leachate was made with 25% C3 leachate and 75% tap water. Additionally, a non-contaminated treatment consisting of tap water (C0) was used as a control. This was done twice during the experimental period, yielding two different sets of contaminated water. We preferred using a maceration solution rather than the leachate from a collection tank to maximize contamination and avoid the risk of using water with a contamination level too low to determine plant tolerance and bioaccumulation in a short time frame, as often occurs in real life situations [39].

**Figure 4:**
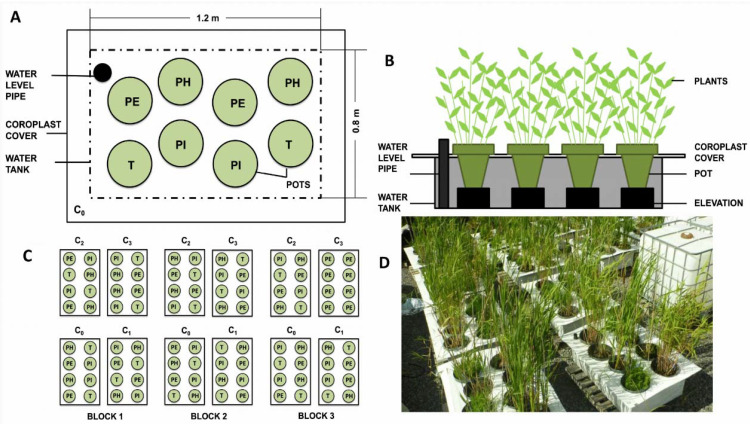
The experimental setup: (**A**) an aerial and (**B**) a side view of a tank, (**C**) the experimental design; (**D**) picture of the experiment. (PH) *Phalaris arundinacea*; (PE) *Phragmites australis* subsp. *australis*; (PI) *Phragmites australis* subsp. *americanus*; (T) *Typha angustifolia*. The four concentrations of leachates are marked with C_0_ (uncontaminated), C_1_–C_3_ (lowest to highest concentration).

The schematics of the experimental setup are shown in Figure 4. The experiment consisted of 12 plastic tanks (1.2 L × 0.8 W × 0.3 H m) that were filled with one of the experimental leachates (C0–C3). Three replicates (i.e., three tanks) were considered for each leachate concentration. A total of 96 plastic pots (30 cm diameter at the top, volume 11 L) were filled with 60% sand and 40% peat mixture. No additional fertilizer was applied, and all nutrients were provided by the peat and the leachate. The pots were planted with one of four macrophyte taxons: *Phalaris arundinacea*, *Phragmites australis* subsp. *australis*, *Phragmites australis* subsp. *americanus*, and *Typha angustifolia*. Two pots of each plant species, for a total of eight pots, were randomly placed in each tank (Figure 1). The pots were elevated with bricks so that the edges of the pots were raised above the edges of the tanks to ensure the surface of the substrate would remain leachate-free. The space between the eight pots in a tank was covered completely by a corrugated plastic sheet to limit precipitation falling into or evaporation from the tank.

The four macrophyte taxons were prepared differently according to the plant propagation type. *P. arundinacea* was planted from seeds purchased from Gloco Inc., Montreal, Quebec, Canada. The *P. australis* subsp. *americanus* and *T. angustifolia* individuals were pre-grown from segments of roots and rhizomes collected from natural populations located near the experimental site. Two-year old *P. australis* subsp. *australis* seedlings were taken from the nursery located at the *Institut de recherche en biologie végétale* (IRBV) field research site in the Montreal Botanical Garden. On 23 and 24 May 2013, *P. arundinacea* was seeded, while for *P. australis americanus*, *T. angustifolia*, and *P. australis australis*, one root segment of each containing a 20 cm-shoot was planted in each pot. Plant species were acclimatized to the experimental conditions 45 days before starting the experiment. During the acclimatization period, each tank was filled with tap water up to 5 cm below the substrate surface (total 160 L per tank). Tap water was added as needed to maintain a constant water level in each tank. The day before starting the experiment, the *P. arundinacea* plants were pruned to keep only 20 shoots per pot to ensure similar plant abundance.

The experiment began on 8 July 2013 and lasted a total of 70 days. At the start of the experiment, the tanks were emptied and refilled with the first set of experimental leachates (C1–C3) and controls with tap water. The volume of the solutions was maintained constant at 160 L in each tank by adding tap water to replace water lost by transpiration. After the first 35 days, the leachates were drained from the tanks and replaced with fresh leachates for an additional 35 days. Since the second batch of leachates was prepared from a new macerated batch of poles, the contaminant concentrations differed considerably from the first leachates (Table 1).

### 4.2. Sampling and Analysis

The C3 leachate was sampled on day one and day 36 of the experiment and analyzed to determine the contaminant concentrations (Table 1). Contaminant concentrations in the C1 and C2 leachates were estimated based on the C3 analysis.

Growth measurements were taken every two weeks (height of stems according to six classes and number of stems) from 8 July and every month from 22 July. To evaluate plant health, stomatal conductance, and leaf chlorophyll content were measured monthly using the Decagon SC-1 Leaf Porometers (Pullman, WA, USA) and the atLEAF Digital Chlorophyll Meter (Wilmington, NC, USA), respectively.

After the first 35 days of exposure, one pot per each plant species in every tank was randomly selected and its plants collected for destructive analysis, which included measuring pollutant concentrations in the plant tissues (above- and belowground biomass separately) and biomass weight analysis. The remaining pots were left in the tanks to extend plant exposure to the contaminants for another 35 days. At the end of the experiment, all remaining plants were collected and analyzed for the same parameters as the first collection.

For the biomass analyses, the above- and belowground plant parts were separated and rinsed with tap water. Belowground parts consisted of roots and rhizomes together. Thirty grams of fresh tissues were collected and kept in a 1 L glass jar stored at 4 °C until tissue analysis. The remaining above- and belowground plant tissues were oven-dried separately at 30 °C (minimum two weeks) until the variation between weights was less than 1% of the previous measurements. The dried plant materials were weighed separately to determine biomass dry weight of the above- and belowground plant parts.

Concentrations of As, Cr, Cu, chlorinated phenols, and PCDD/F in the C3 leachates and the plant tissues were determined by an accredited analytical laboratory (AGAT, Montreal, QC, Canada). Acid extractable As, Cr, and Cu were determined using inductively coupled plasma mass spectrometry (ICP-MS, Agilent, Santa Clara, CA, U.S.A.) (Report MA.200-Mét. 1.2, Rev. 5, [75]). Determination of chlorinated phenols was conducted using the gas chromatography assay coupled to a mass spectrometer (GC-MS, Agilent) after derivation with acetic anhydride (Report MA. 400-Phé 1.0, Rev. 3, [75]). PCDD/F was determined by high resolution gas chromatography coupled to a high-resolution mass spectrometer (HRGC/HRMS, Thermo fisher) (Report MA. 400-D.F. 1.1, Rev. 1, [75]).

All analyses were performed according to certified reference materials and following quality control/quality assurance (QC/QA) procedures. The recovery values for the As, Cr, and Cu analysis were in the range of 70–130% for the solutions and 50–150% for the plant tissue samples. The values for the analysis of chlorinated phenols were between 20 and 110%.

Statistical analyses were performed using JMP statistical software (SAS Institute Inc., Cary, NC, USA). The effects of lixiviate leachate contamination level on plant parameters and concentration of contaminants in plant tissues were tested using a mixed-model ANOVA with the block factor as a random variable after the assessments of normality and homoscedasticity had been verified. In the many cases where the concentrations of contaminants in the plant tissues were below the detection level for a large proportion of the replicates, no statistical analyses were performed. When significant main effects were found (*p* < 0.05), we compared means using Tukey’s HSD multiple comparison test.

It must be noted that our emphasis was on testing differences in biomass and tissue contents between contamination levels within each plant species, rather than to test statistical differences between species. As the plant species were not propagated in the same manner, they had different biomass at the start of the experiment along with inherent differences in the time required to reach maturity; comparisons between plant species based on a short-term experiment must be interpreted with care. We preferred to limit our interpretation of these differences to the qualitative level.

## 5. Conclusions

Overall, our results showed that *P. arundinacea*, *P. australis* subsp. *australis*, *P. australis* subsp. *americanus*, and *T. angustifolia* had the ability to survive and grow well under experimental conditions analogous to a subsurface flow treatment wetland receiving a contaminated leachate with wood preservatives. We found no evidence that bioaccumulation of the toxins in the aboveground portions of the plants was sufficient to allow removal using phytoextraction and plant harvesting. In the context of a subsurface constructed wetland treating a contaminated leachate, a large part of the retention would be the result of adsorption in the substrate.

However, the studied species demonstrated some capacity to accumulate As and chlorinated phenols in their belowground parts and might have been able to bioaccumulate Cr and Cu at higher exposure concentrations, thus contributing to immobilization of the toxins. In addition, these plants may potentially stimulate bacterial degradation of chlorinated phenols. For example, Kurzbaum et al. [76] showed greater microbial degradation of PCPs in the rhizosphere of *Phragmites australis* when compared with unplanted TW mesocosms. Plants may also contribute to phytostabilization of contaminants by reducing current velocity, stabilizing the sediment, releasing oxygen, and enhancing the physical, chemical, and microbial processes [26,33,77]. Other than in the context of treatment wetlands, the plant species tested may be used for phytostabilization and restoration of natural wetlands contaminated with wood preservatives. *Phragmites australis* subsp. *americanus* and *T. angustifolia* are particularly promising species considering their high biomass production and remediation potential.

Several aspects require further study. For example, an increase in the contaminant concentration of the leachates or in the exposure duration would be required to fully assess the efficacy of the approach and better understand the possible interactions between the metal(loid)s and chlorinated phenols and their impacts on toxicity and bioaccumulation. The root microbiomes of the plants exposed to such concentrations of contaminants could also be analyzed to determine the role of microorganisms in the fate of organic pollutants.

## Figures and Tables

**Table 1:** Concentration of pollutants (µg/L) in C1–C3 leachates on day 1 and day 36 of the experiment. (MCP) monochlorophenols, (DCP) dichlorophenol, (TCP) trichlorophenol, (TeCP) tetrachlorophenol, (PCP) pentachlorophenol, (DL) detection limit.

Pollutants		Concentration (µg/L)				
	Day 1			Day 36		
	C1	C2	C3	C1	C2	C3
MCP	2	5	10	<DL	<DL	<DL
DCP	2	4	9	<DL	<DL	<DL
TCP	<DL	<DL	<DL	<DL	<DL	<DL
TeCP	105	210	419	2	5	10
PCP	1075	2150	4300	20	40	81
Arsenic	83	165	330	223	445	890
Chromium	10	20	40	20	40	80
Copper	25	50	100	100	200	400
Sum of dioxins	2.36	4.72	9.44	0.45	0.9	1.8
Sum of furans	2.13	4.25	8.5	0.22	0.44	0.89
pH	7.1	7.5	7.4	6.9	7.0	7.3

## Supplementary Materials

The following are available online at https://www.mdpi.com/2223-7747/9/12/1774/s1. Table S1: Plant parameters of the four macrophyte species exposed to the contaminated leachates (C0, C1, C2 and C3) after 35 days and 70 days. Table S2a: *P*-value of the Anova testing the difference in arsenic concentration in belowground plant tissue in relation to contamination levels, for each species taken individually, and for each of the two sampling dates. The concentrations are considered significant for *p* < 0.05. Table S2b: Results (groups) of the post-hoc Tukey’s test (*p* < 0.5) performed for species-date that showed significant differences in arsenic concentration in belowground tissue according to the Anova. Table S3: The chlorinated phenol concentrations (mg/kg DW) in the belowground dry biomass of the four macrophyte species exposed to the contaminated leachates (C1, C2 and C3) after 35 days and 70 days.
