# Supplementary material for: Macrophyte Potential to Treat Leachate Contaminated with Wood Preservatives: Plant Tolerance and Bioaccumulation Capacity
Source: Plants (Basel). 2020 Dec 14;9(12):1774. doi: 10.3390/plants9121774 (PMC7765096; doi:10.3390/plants9121774)
Supplement: Supplementary file 1 [file plants-09-01774-s001.pdf]

Table S1. Plant parameters of the four macrophyte species exposed to the contaminated leachates (C0, C1, C2 and C3) after 35 days and 70 days.

Species / Contamination level	Day	*Phalaris arundinacea* C0	C1	C2	C3	*Phragmites australis australis* C0	C1	C2	C3
Aboveground biomass (g DW)	35	8.1 (3.8)	4.2 (2.8)	2.4 (0.5)	3.1 (1.5)	43.0 (21.1)	23.6 (0.7)	17.7 (9.7)	21.1 (36.5)
	70	29.5 (2.2)	13.2 (3.2)	17.1 (2.7)	13.4 (14.5)	64.6 (20.5)	27.2 (23.9)	33.1 (25.36)	58.2 (25.5)
Belowground biomass (g DW)	35	15.5 (14.8)	4.3 (0.9)	9.2 (5.0)	3.1 (4.2)	32.5 (17.7)	67.8 (40.4)	29.6 (8.1)	19.9 (21.9)
	70	39.2 (4.6)	26.2 (6.9)	40.2 (16.9)	35.1 (8.4)	62.0 (9.1)	39.5 (32.1)	83.5 (60.5)	65.1 (26.7)
Number of shoots	35	12.5 (5.8)	81 (18.4)	71 (15.5)	64 (30.7)	18 (6.6)	13 (3.6)	9.3 (5.5)	9.7 (5.0)
	70	107 (1.2)	91.6 (25)	84.3 (6.1)	107 (26.8)	18 (0)	11..3 (8.6)	16.3 (1.5)	21.3 (3.2)
Height of shoots (cm)	35	18 (2.5)	20 (5.1)	18.2 (1.6)	18.3 (0.7)	69 (8.6)	59 (12.2)	41 (16.6)	52 (18.1)
	70	35.6 (2.8)	31.9 (6.1)	34.7 (0.5)	34.3 (3.2)	69 (14)	50 (38.8)	68 (25)	66 (8.7)
Chlorophylle (Spad units)	35	37.0 (1.4)	36.7 (3.5)	34.4 (1.1)	39.1 (7.2)	50.3 (5.1)	44.2 (6.8)	43.8 (10.8)	48.7 (3.8)
	70	33.4 (0.2)	31.5 (5.2)	30.2 (8.4)	31.3 (7.2)	44.9 (2.8)	39.2 (1.3)	38.4 (6.5)	42.9 (7.0)
Stomatal conductance (mmol/m2s)	35	197 (31)	241 (95)	265 (26)	223 (63)	411 (81)	381 (78)	324 (157)	446 (10)
	70	200 (76)	268 (57)	197 (12)	167 (128)	382 (86)	331 (103)	375 (44)	326 (51)

Species / Contamination level	Day	*Phragmites australis americanus* C0	C1	C2	C3	*Typha angustifolia* C0	C1	C2	C3
Aboveground biomass (g DW)	35	61.9 (12.1)	54.9 (11.8)	78.6 (27.1)	51.5 (28.4)	59.3 (5.3)	63.5 (20.4)	59.2 (6.8)	60.3 (14.7)
	70	108.3 (44.5)	62.8 (20.0)	75.3 (39.8)	85.8 (30.7)	109.0 (28.0)	60.6 (17.9)	73.2 (11.6)	67.0 (19.3)
Belowground biomass (g DW)	35	50.8 (15.5)	42.5 (19.9)	73.1 (10.9)	51.8 (9.9)	85.3 (17.4)	120.6 (51.9)	154.4 (17.1)	148.7 (13.5)
	70	108.2 (46.3)	68.3 (12.3)	72.9 (34.8)	105.4 (65.5)	271.5 (106.0)	174.0 (66.9)	201.3 (61.3)	215.6 (30.3)
Number of shoots	35	23 (7.8)	22 (1.5)	27 (11.6)	29 (8.0)	5.3 (2.1)	4.7 (0.6)	8.3 (1.5)	6.3 (1.5)
	70	25 (8.6)	26 (9.3)	28 (1.2)	32 (11.8)	8.7 (5.1)	5.3 (2.1)	7.0 (2.0)	7.0 (2.6)
Height of shoots (cm)	35	58 (17.2)	75 (12.5)	70 (14.2)	78 (6.9)	103 (26)	121 (15)	101 (3.8)	106 (8.8)
	70	82 (16.6)	63 (9.9)	88 (10.6)	74 (2.0)	127 (7.0)	113 (13)	112 (18)	120 (3)
Chlorophylle (Spad units)	35	43.2 (3.6)	44.8 (1.7)	39.4 (7.6)	39.7 (0.5)	62.5 (6.4)	59.7 (1.4)	60.1 (2.3)	53.4 (4.4)
	70	37.5 (2.5)	35.6 (3.8)	36.7 (2.6)	36.1 (1.5)	55.8 (4.3)	55.4 (8.3)	54.1 (8.0)	53.0 (1.5)
Stomatal conductance (mmol/m2s)	35	371 (65)	337 (52)	330 (29)	375 (87)	439 (105)	366 (125)	319 (61)	330 (37)
	70	322 (2.2)	228 (63)	335 (50)	241 (52)	220 (76)	235 (91)	270 (73)	232 (52)

Table S2a. P-value of the Anova testing the difference in arsenic concentration in belowground plant tissue in relation to contamination levels, for each species taken individually, and for each of the two sampling dates. The concentrations are considered significant for $p < 0.05$.

	Phalaris arundinacea	*Phragmites australis australis*	*Phragmites australis americanus*	*Typha angustifolia*
After 35 days	0.0605	0.0423*	0.0605	0.0552
After 70 days	0.1988	0.0379*	0.0347*	0.0221*

Table S2b. Results (groups) of the post-hoc Tukey's test ($p < 0.5$) performed for species-date that showed significant differences in arsenic concentration in belowground tissue according to the Anova.

	Contamination level	*Phalaris arundinacea*	*Phragmites australis australis*	*Phragmites australis americanus*	*Typha angustifolia*
After 35 days	C1	-	c	-	-
	C2	-	b	-	-
	C3	-	a	-	-
After 70 days	C1	-	b	b	b
	C2	-	a	b	b
	C3	-	a	a	a

Table S3. The chlorinated phenol concentrations (mg/kg DW) in the belowground dry biomass of the four macrophyte species exposed to the contaminated leachates (C1, C2 and C3) after 35 days and 70 days.

Species	Days	Conc	Monochlorophenol				Dichlorophenol				Trichlorophenol				Tetrachlorophenol				Pentachlorophenol			
			Replicate #			Mean	Replicate #			Mean	Replicate #			Mean	Replicate #			Mean	Replicate #			Mean
			1	2	3		1	2	3		1	2	3		1	2	3		1	2	3	
Phalaris	**35**	**C1**	-	-	-	0	-	-	-	0	-	-	-	0	-	-	-	0	-	-		0
arundinacea		**C2**	-	-	-	0	-	-	-	0	0,2	-	-	0,07	-	-	-	0	-	-	-	0
		**C3**	-	-	-	0	-	-	-	0	-	-	-	0	-	-	-	0	-	-	-	0
	**70**	**C1**	-	-	-	0	-	-	-	0	-	0,2	-	0,07	-	-	-	0	-	-	-	0
		**C2**	-	-	-	0	-	-	-	0	-	-	-	0	-	-	-	0	-	-	-	0
		**C3**	-	0,3	0,8	0,37	-	0,1	0,2	0,10	0,1	0,2	0,1	0,13	-	-	-	0	-	-	-	0
															-							
Phragmite	**35**	**C1**	-	-	-	0	-	-	-	0	-	0,9	-	0,30	-	-	-	0	-	-	-	0
australis		**C2**	-	-	-	0	-	-	-	0	-	-	0,1	0,03	-	-	-	0	-	-	-	0
australis		**C3**	-	-	-	0	-	-	-	0	-	0,2	-	0,07	-	-	-	0	0,2	-	-	0,07
	**70**	**C1**	-	-	-	0	-	-	-	0	-	-	-	0	-	-	-	0	-	-	-	0
		**C2**	0,1	0,2	-	0,10	-	-	-	0	1,1	0,4	3,1	1,53	-	-	0,5	0,17	-	0,1	0,2	0,1
		**C3**	0,2	0,9	0,5	0,53	-	0,8	-	0,27	0,2	1,5	0,3	0,67	-	-	0,1	0,03	-	0,1	0,2	0,1
Phragmite	**35**	**C1**	-	-	-	0	-	-	-	0	-	1,9	4,1	2,00	-	0,3	1	0,43	-	-	0,2	0,07
australis		**C2**	-	-	-	0	-	-	-	0	-	2,1	0,8	0,97	-	0,6	0,1	0,23	-	0,3	-	0,1
americanus		**C3**	-	-	-	0	-	-	-	0	0,3	2	1,5	1,27	-	0,5	0,6	0,37	-	0,7	0,6	0,43
	**70**	**C1**	-	-	-	0	-	-	-	0	0,3	0,5	0,4	0,40	0,1	-	0,1	0,07	-	-	-	0
		**C2**	-	0,1	-	0,03	-	0,1	-	0,03	0,5	4,2	0,1	1,60	-	0,6	-	0,20	-	0,3	-	0,1
		**C3**	1,5	0,8	0,2	0,83	1,1	0,3	0,3	0,57	3,5	1,9	3	2,80	0,4	0,2	0,5	0,37	0,8	0,2	0,5	0,50
Typha	**35**	**C1**	-	-	-	0	-	-	-	0	1,1	-	-	0,37	0,2	-	-	0,07	-	-	-	0
angustifolia		**C2**	-	-	-	0	-	-	-	0	1,4	0,2	0,3	0,63	0,2	-	-	0,07	-	-	-	0
		**C3**	-	-	-	0	-	-	-	0	0,6	1,4	0,7	0,90	-	0,4	0,6	0,33	-	0,3	0,8	0,37
	**70**	**C1**	-	-	-	0	-	-	-	0	3	0,2	-	1,07	0,3	-	-	0,10	-	-	-	0
		**C2**	-	-	0,5	0,17	-	-	0,1	0,03	0,2	0,7	1,1	0,67	-	0,2	0,2	0,13	0,1	0,5	0,2	0,27
		**C3**	0,7	-	1,2	0,63	0,1	-	0,7	0,27	0,3	0,1	1,2	0,53	-	-	0,4	0,13	-	0,1	1	0,37
